# Case Report: Single Dose Anti-PD1 in a Patient With Metastatic Melanoma and Cardiac Allograft

**DOI:** 10.3389/fimmu.2021.660795

**Published:** 2021-03-22

**Authors:** Amy E. Goodman, Lilit Karapetyan, Melissa Pugliano-Mauro, Joshua E. Levenson, Jason J. Luke

**Affiliations:** ^1^Hillman Cancer Center, UPMC, Pittsburgh, PA, United States; ^2^Department of Medicine, University of Pittsburgh, Pittsburgh, PA, United States; ^3^Department of Dermatology, University of Pittsburgh, Pittsburgh, PA, United States; ^4^Center for Cardio-Oncology, UPMC, Pittsburgh, PA, United States

**Keywords:** immunotherapy, genomics, biomarkers, immune-related adverse events, transplant, case report

## Abstract

**Background:**

Immune-checkpoint inhibition has improved outcomes in metastatic melanoma. However, limited data describes the safety and efficacy of this treatment in the setting of cardiac allograft. Emerging translational and clinical evidence suggests that the majority of the benefit from these therapies is driven by the initial dose(s), and that attenuated dosing schedules may be as effective as continuous treatment.

**Case presentation:**

We present a case vignette of a cardiac transplant recipient with metastatic melanoma who experienced six months of clinical benefit after one dose of pembrolizumab and did not suffer allograft rejection.

**Conclusion:**

This case adds to the current available literature on the administration of checkpoint inhibitors in patients with cardiac allografts. Further, it explores potential markers of immunotherapy response and supports the potential of shorter or individualized immune-checkpoint blockade dosing strategies.

## Introduction

Immune checkpoint inhibition therapy has fundamentally changed the treatment of metastatic melanoma. However, given the inherent risk of allograft rejection, there is limited data on the safety and efficacy of these treatments in transplant recipients. Here, we describe a patient with metastatic melanoma in the setting of cardiac allograft who was treated with one dose of pembrolizumab. His tumor was notable for *NF1* mutation, negative PD-L1, and high tumor mutational burden. Following the single dose, he experienced clinical benefit for six months with improved symptomatology, downtrending LDH and stable imaging. He unfortunately eventually passed away due to disease progression, but he did not have evidence of allograft rejection. We discuss the recent literature supporting response after one or a few doses of immunotherapy, as well as markers used to predict treatment response. This case emphasizes that longer or perhaps even individualized dosing schedules may be possible in the future of immunotherapeutics.

## Case Report

A man in his late 60s with a history of orthotopic cardiac transplant in 2002 was diagnosed 17 years post-transplant with metastatic melanoma of unknown primary. The transplant was performed secondary to non-ischemic cardiomyopathy in the context of cytomegalovirus infection. The graft maintenance strategy initially included mycophenolic acid plus tacrolimus, however was modulated several times due to complications including calcineurin-induced thrombotic thrombocytopenic purpura, seizures, and immune-suppression related malignancies including stage IIA colorectal cancer, melanoma *in situ* of the right cheek and cutaneous squamous cell carcinoma *in situ*. The previous melanoma lesion was treated with wide local excision and close observation, without obvious evidence of recurrence. Additional medical comorbidities included chronic kidney disease, recurrent venous thrombosis, and pulmonary embolism associated with positive lupus anticoagulant. He was eventually on sirolimus alone, which was the regimen at the time of melanoma diagnosis.

**Figure 1:**
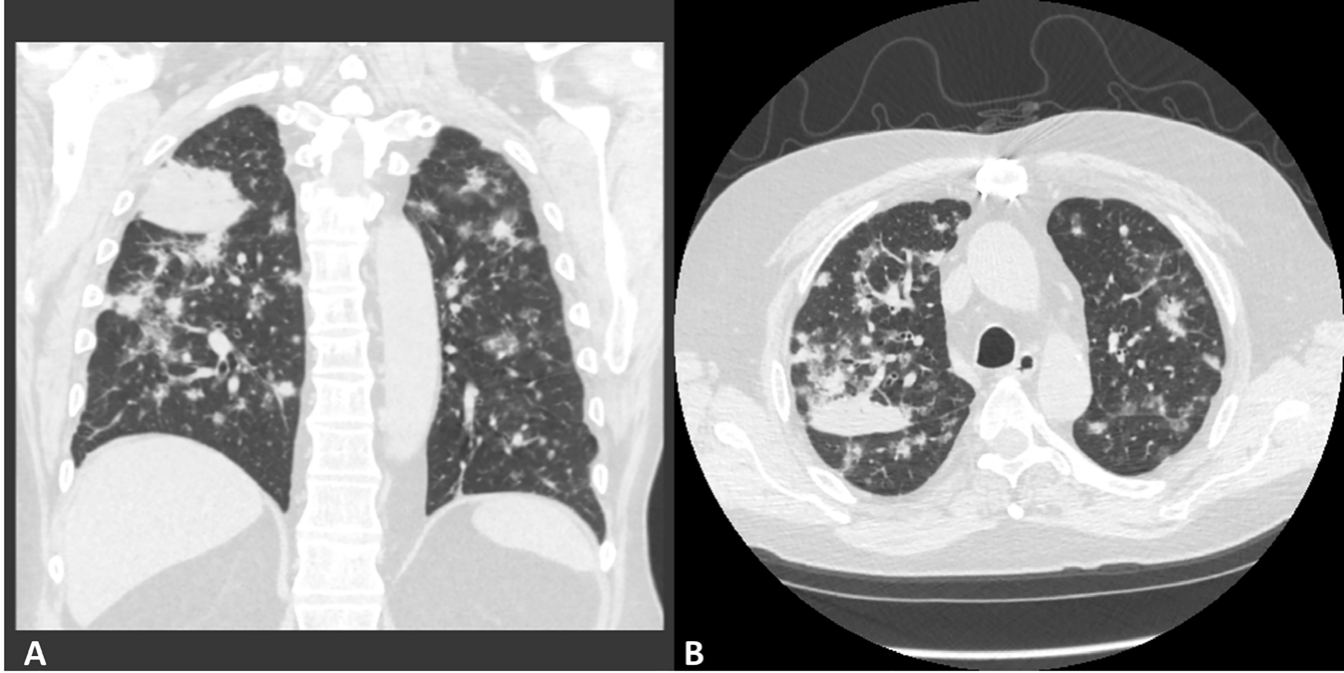
Coronal **(A)** and axial **(B)** representative images from the patient’s pre-treatment CT scan.

In October 2019, the patient developed increasing dyspnea. Computed tomography (CT) of the chest demonstrated diffuse nodular ground-glass infiltrates and widespread nodularity bilaterally (**Figure 1**). Bronchoscopy with fine needle aspiration demonstrated rare atypical cells positive for HMB45 and SOX10, suggestive of melanoma.

The patient presented to medical oncology in December 2019. Staging PET/CT demonstrated extensive disease in the lung and multifocal osseous metastases. A percutaneous CT-guided biopsy of the right upper lobe lung mass confirmed metastatic melanoma expressing HMB45, S100 and SOX10. Next generation sequencing revealed wild type status of *BRAF* and *NRAS*, stable microsatellite status, and mutations in: *NF1* K476, *RAC1* P29S, *TERT* C229T, *EZH2* Y646N, *CBL* Y371N, and *FANCA* Q1044. The specimen also showed copy number loss in *MTOR*, *ERBB4*, *ROS1/ESR1*, *CHEK1/CBL*, and *SMAD4*. Tumor mutational burden (TMB) was 29.0 mutations/Mb.

Options for treatment including immune-checkpoint inhibition, cytotoxic chemotherapy, and off-label use of MEK inhibitors were considered. Cytotoxic chemotherapy was deferred given the poor general toxicity/benefit ratio. The risk of graft rejection and lack of clear evidence of safety for checkpoint inhibition in transplant patients nominated MEK inhibition as the initial treatment approach. Trametinib was initiated on January 6, 2020. Simultaneously sirolimus dosing was minimized with a new goal level of 6-8 ng/mL (from previous of 10-12 ng/mL). Three weeks after initiating therapy the patient experienced clinical decline with increased shortness of breath, hypoxia, and extreme fatigue felt to be due to worsening of the underlying metastatic melanoma.

**Figure 2:**
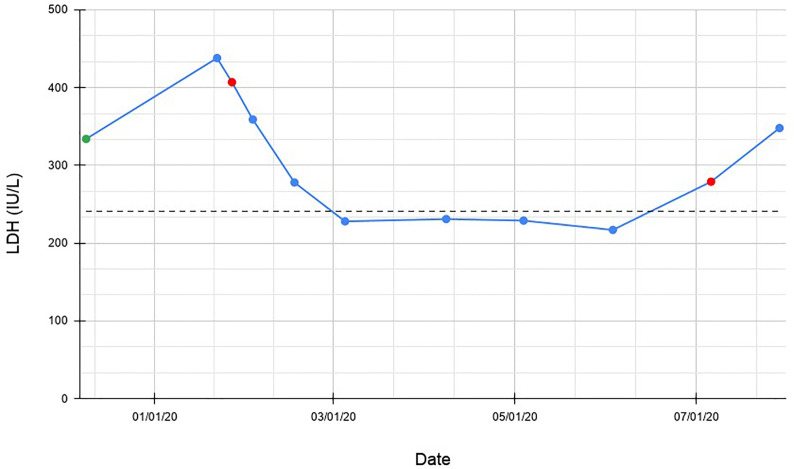
Change in serum LDH over time. The green data point represents initial diagnosis and initiation of treatment with trametinib. Red data points represent dates that pembrolizumab was administered. Dotted line represents upper limit of normal LDH (241 IU/L).

**Figure 3:**
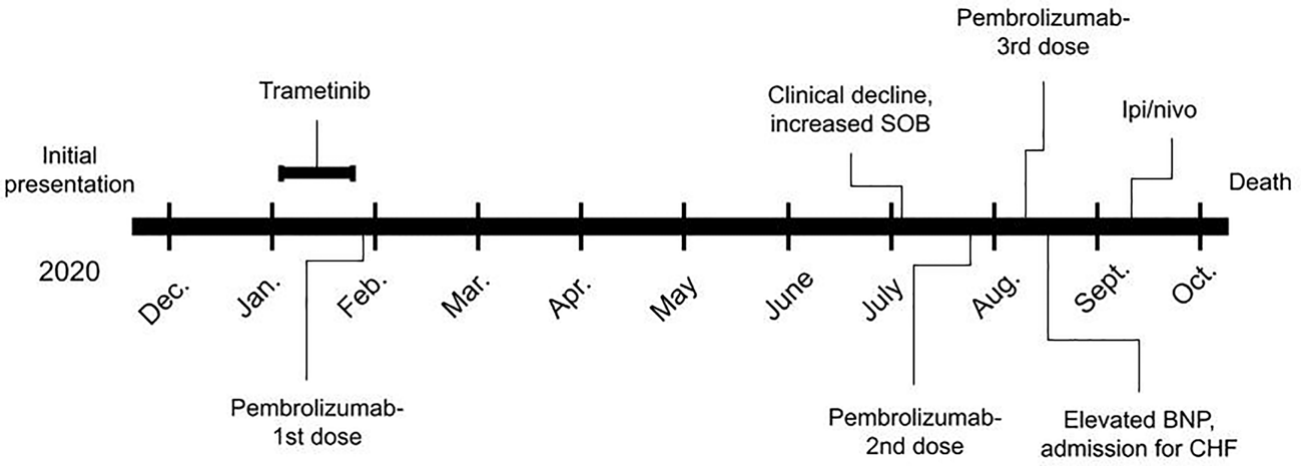
Timeline representation of the case.

Although tumor expression of PD-L1 was 0%, response to immune-checkpoint inhibition was felt to have a potential for success given the extreme TMB at 87.5 percentile of melanoma samples. Additionally, the presence of the *NF1* mutation and the location of the previous melanotic lesion on the cheek raised the possibility of this melanoma being of desmoplastic lineage, a sub-type associated with extremely high rates of response to anti-PD1 (1). Risks and benefits were reviewed, including the extreme possibilities of cardiac allograft rejection and death versus treatment response and long-term survival for melanoma. The patient chose to pursue immunotherapy and was given one dose of anti-PD1 with pembrolizumab 200 mg intravenous infusion on January 27, 2020. Follow up bloodwork revealed a precipitous drop in the serum lactic dehydrogenase (LDH), eventually below the upper limit of normal (**Figure 2**). Over the next three weeks the patient demonstrated noticeable improvement in shortness of breath and energy level. While an adverse effect from the initial MEK inhibitor therapy such as pneumonitis was considered, it seemed less likely given the improvement in clinical status with no intervention such as steroids and the long half-life of trametinib of approximately 4.5 days. As such, the drug would have still been at clinically significant levels for three to four weeks following discontinuation, even while the patient was clinically improving. The rapid improvement upon initiation of anti- PD1 therapy further supported that the initial decline was due to worsening of the melanoma. Further pembrolizumab was held in an attempt to minimize allograft rejection risk. Re-staging CT scans were performed three months later demonstrating slight improvement in the right upper lobe and otherwise stable lesions compared with the baseline PET/CT. Stable disease was maintained for six months until he began to clinically decline again, and CT suggested progressive disease. Two further doses of pembrolizumab 200 mg were given three weeks apart, six months from his first dose. His cardiac allograft status was monitored with serum B-natriuretic peptide (BNP) levels, troponin I levels, and echocardiograms. His BNP became elevated above baseline after his third dose of pembrolizumab, and he was admitted and treated for heart failure. Cardiology did not feel the patient was experiencing graft rejection, but rather right heart failure due to mild volume overload and the consequences of extensive disease burden. Unfortunately due to his progressive disease he continued to decline, and despite attempts at salvage therapy with ipilimumab and nivolumab as well as stereotactic radiosurgery for new brain metastases, he transitioned to hospice in October 2020 and passed away. A graphical representation of the case is shown in **Figure 3**.

## Discussion

Melanomas are grouped into four major subtypes based on mutational profile of *BRAF*, *RAS, NF1*, or triple wild-type (2). *NF1* is a tumor suppressor that exerts negative feedback on the RAS/MAPK pathway. Preclinical literature and some case reports suggest that MEK inhibition may inhibit NF1 induced MAPK signaling, leading to our choice to pursue this as an initial treatment strategy (3). Additionally, the presence of the *NF1* mutation raised the possibility that this melanoma was of desmoplastic subtype, a less common presentation with a high frequency of *NF1* mutations. Desmoplastic melanomas have been described to have a high expression of PD-L1 and frequently high TMB, more than twice that of other melanoma subtypes. Similarly response rates to anti-PD1/L1 for desmoplastic melanomas have been described as approximately 70% versus ~40% for cutaneous melanomas more broadly [1]. The strongest predictors of response to anti-PD1 in melanoma include PD-L1 expression, interferon-γ associated gene expression, and high TMB (4, 5). This melanoma demonstrated 0% PD-L1 expression but an extremely high TMB emphasizing the limited interaction between these biomarkers and the importance of neoantigenicity.

Risk of organ graft rejection led to the exclusion of transplant recipients from immunotherapy clinical trials and limited data are available to judge the safety and efficacy of immune-checkpoint blockade in this population. A pooled analysis of 64 patients suggested graft rejection due to checkpoint inhibition of 41%, with 20% (n=5) specifically in cardiac allografts. Rates of rejection appeared to be higher due to anti-PD1 than anti-CTLA4 with efficacy consistent with non-transplant populations. No clear correlation was observed between the number of doses of checkpoint inhibitor therapy given or time from transplant and rate of rejection. In one case, a renal transplant recipient achieved complete response after two months of pembrolizumab and continued to receive treatment. He had no evidence of graft rejection until eight months of treatment, at which time he developed acute cell-mediated rejection (6). This raises the possibility that minimizing exposure to checkpoint blockade may be a priority for patients with concurrent organ grafts.

To this effect, we attempted to balance the risk of graft rejection with anti-cancer treatment benefit. Neoadjuvant clinical trials in melanoma have suggested potential for long-term immune responses and clinical benefit with only a single or a few doses of immunotherapy. Huang *et al*. demonstrated in a neoadjuvant study that a single dose of anti-PD1 could induce major pathologic response by three weeks, which was associated with disease-free survival beyond two years. A significant increase in Ki67+ CD8 T-cells was seen in the peripheral blood of responding subjects, which peaked at seven days post treatment. This suggests that the tumor may already have infiltration of exhausted T cells which became quickly re-invigorated upon administration of PD1 blockade (7). In the metastatic setting, a pilot study has suggested that the first two doses of ipilimumab with nivolumab might be used to predict treatment response. Patients who had favorable response after two doses transitioned to maintenance nivolumab whereas progressing patients continued through four doses. Strikingly, all patients with clinical benefit manifested this on imaging following two doses (8). Evolving evidence also supports the contention that immune-checkpoint antibodies might be given less frequently than originally studied due to high receptor saturation even after one dose (9). Our patient exhibited stable disease for six months from the first dose of pembrolizumab and it remains unclear whether continued dosing is required to avoid eventual disease progression or whether tumors progress despite continued treatment (10). It seems unlikely that this outcome could be attributed to the initial trial of MEK inhibition or would have occurred even in the absence of treatment, given the patient’s initial decline, the lack of support in the clinical literature surrounding MEK inhibition for NF1 mutant melanoma, and the rapid improvement seen following the single anti-PD1 dose. This case emphasizes that longer, and perhaps individualized, dosing intervals might be considered as a priority area of investigation to optimize immunotherapy.

At times difficult decisions must be made between a patient, their family and the treatment team when managing multiple life threatening conditions. The balance of risk and benefit can be a fine one which evolves over the course of treatment and requires constant re-evaluation. More data on potential markers to predict response to therapy or the ability to discontinue therapy should be high priorities to the field. Clinical investigations into lengthening or individualizing dosing intervals are an area of high unmet need and would be invaluable toward optimal management in cases such as this.

## Patient Perspective

The patient and his wife were fully engaged throughout the treatment process. We had multiple in-depth conversations regarding the potential risks and benefits if immune checkpoint inhibitor therapy in the setting of organ transplant. After the patient passed away, his wife was happy to agree to publication of this case report in hopes of furthering medical knowledge in this area. Written informed consent was obtained from her as next of kin.

## Data Availability Statement

The original contributions presented in the study are included in the article/supplementary material. Further inquiries can be directed to the corresponding author.

## Ethics Statement

Ethical review and approval was not required for the study on human participants in accordance with the local legislation and institutional requirements. The patients/participants provided their written informed consent to participate in this study. Written informed consent was obtained from the individual(s) for the publication of any potentially identifiable images or data included in this article.

## Author Contributions

AG, LK, MP-M, JEL, and JJL all participated in the management of this case. AG and JJL were in charge of manuscript drafting and data collection. All authors contributed to the article and approved the submitted version.

## Funding

JJL acknowledges Department of Defense Career Development Award (W81XWH-17-1-0265), the Sy Holzer Endowed Immunotherapy Research Award, and the Hillman Fellowship for Innovative Cancer Research.

## Conflict of Interest

JJL declares Scientific Advisory Board: (no stock) 7 Hills, Spring bank (stock) Actym, Alphamab Oncology, Arch Oncology, Kanaph, Mavu, Onc.AI, Pyxis, Tempest. Consultancy with compensation: Abbvie, Array, Bayer, Bristol-Myers Squibb, Checkmate, Cstone, Eisai, EMD Serono, KSQ, Janssen, Merck, Mersana, Nektar, Novartis, Pfizer, Regeneron, Ribon, Rubius, Silicon, Tesaro, TRex, Werewolf, Xilio, Xencor. Research Support: (all to institution for clinical trials unless noted) AbbVie, Agios (IIT), Array (IIT), Astellas, Bristol-Myers Squibb (IIT & industry), Corvus, EMD Serono, Immatics, Incyte, Kadmon, Macrogenics, Merck, Moderna, Nektar, Numab, Replimmune, Rubius, Spring bank, Synlogic, Takeda, Trishula, Tizona, Xencor. Travel: Pyxis. Patents: (both provisional) Serial #15/612,657 (Cancer Immunotherapy), PCT/US18/36052 (Microbiome Biomarkers for Anti-PD-1/PD-L1 Responsiveness: Diagnostic, Prognostic and Therapeutic Uses Thereof).

The remaining authors declare that the research was conducted in the absence of any commercial or financial relationships that could be construed as a potential conflict of interest.
